# Draft Genome Sequence of Aeromonas caviae Strain A1-2, a Potential Plant Growth-Promoting Rhizospheric Bacterium

**DOI:** 10.1128/mra.00983-22

**Published:** 2022-11-09

**Authors:** Olubukola Oluranti Babalola, Victor Funso Agunbiade, Ayomide Emmanuel Fadiji

**Affiliations:** a Food Security and Safety Focus Area, Faculty of Natural and Agricultural Sciences, North-West University, Mmabatho, South Africa; University of Arizona

## Abstract

The genomic analysis of the plant growth-promoting rhizospheric Aeromonas caviae strain A1-2, which was isolated from a maize plant in Northwest Province, South Africa, is presented in this paper. Aeromonas caviae strain A1-2 demonstrates its potential to promote plant growth and enhance the tolerance of maize plants to drought stress.

## ANNOUNCEMENT

Global demand for agricultural products is increasing due to the increasing human population. However, several studies have highlighted the challenges associated with the impacts of drought stress and indiscriminate fertilizer applications on crop plants, raising public concerns about food security and agricultural sustainability (1–3). Ecologically friendly strategies are required to avoid the problems associated with chemical fertilizer use and chemical drought mitigation strategies.

Soil samples were collected from maize (*Zea may* L.) plantations at the teaching and research farm of the North-West University, South Africa (25°47′25.24056″S, 25°37′8.17464″E), using the methods described by Sha’arani et al. (4). A pure rhizospheric bacterium was isolated from 10 g of soil tightly adhering to the root of a sorghum plant by serial dilution and plating on Luria-Bertani (LB) agar plates as described by Ahmad et al. (5) and Majeed et al. (6). After subculturing, the single colony was confirmed through growth on LB agar and through morphological and biochemical observations of the strain. Aeromonas caviae strain A1-2 showed positive results in *in vitro* tests for plant growth-promoting traits such as indole-3-acetic acid production (7), nitrogen fixation (5), phosphate production (8), 1-aminocyclopropane-1-carboxylate (ACC) deaminase production (9), ammonia production (10), and siderophore production (5).

The genomic DNA from the pure culture on solid agar was extracted using a soil microbe extraction kit (Zymo Research, USA). The genome sequence of Aeromonas caviae strain A1-2 was obtained using the Illumina Nextera DNA Flex library preparation kit for library preparation. The sequence reads were generated using the Illumina NextSeq 550 system (2 × 150 bp) at the Inqaba Biotechnology Industries, South Africa. The sequence was visualized using KBase (11), which yielded 10,038,572 bp of raw data from the sequencing run. Using FastQC version 1.0.4 (12), the quality of the reads was examined. Trimmomatic version 1.2.14 (13) was used to filter out the adapter regions and low-quality reads from the data. The genome assembly was created using SPAdes version 1.2.4 (14), which yielded a total genome size of 4,237,144 bp, with 182 contigs, a GC content of 61.9%, an *N*_50_ value of 40,856 bp, and an *L*_50_ value of 32. This file was then used to perform BLAST analysis against the Genome Taxonomy Database (GTDB) with GTDB-Tk version 1.7.0 (15). All software was run with default settings unless otherwise specified.

The annotation for the prediction of gene functions was carried out employing the freely accessible NCBI Prokaryotic Genome Annotation Pipeline (PGAP) (16). The results revealed a total of 4,023 genes, with 3,949 coding DNA sequences (CDSs), 39 pseudogenes, and 79 noncoding sequences (6 rRNAs, 67 tRNAs, and 6 noncoding RNAs [ncRNAs]).

### Data availability.

The whole-genome sequence of Aeromonas caviae strain A1-2 has been deposited in DDBJ/ENA/GenBank under the accession number JANSJY000000000. The version described in this paper is version JANSJY010000000. The raw reads are available under the BioProject accession number PRJNA870035 and the BioSample accession number SAMN30346362; the sequence data obtained in this work have been deposited in the NCBI Sequence Read Archive (SRA) under the accession number SRR21084892.
